# Chromosome Unipolar Division and Low Expression of *Tws* May Cause Parthenogenesis of Rice Water Weevil (*Lissorhoptrus oryzophilus* Kuschel)

**DOI:** 10.3390/insects12040278

**Published:** 2021-03-24

**Authors:** Pengcheng Wang, Fangyuan Yang, Zhuo Ma, Runzhi Zhang

**Affiliations:** 1 Key Laboratory of Zoological Systematics and Evolution, Institute of Zoology, Chinese Academy of Sciences, Beijing 100101, China; wangpengcheng@ioz.ac.cn (P.W.); yangfy@ioz.ac.cn (F.Y.); mazhuo@ioz.ac.cn (Z.M.); 2 College of Life Sciences, University of Chinese Academy of Sciences, Beijing 100049, China; 3 Department of Entomology, Guizhou University, Guiyang 550025, Guizhou, China

**Keywords:** parthenogenesis, *Lissorhoptrus oryzophilus*, meiosis, *Tws*, *REC8*

## Abstract

**Simple Summary:**

Rice water weevil (RWW), *Lissorhoptrus oryzophilus* (Kuschel) (Coleoptera: Brachyceridae), is one of the main pests of rice. In 1959, the parthenogenetic RWW was first reported in California and invaded Asia in 1978. It is known that sexually reproducing RWW is a diploid organism, while parthenogenetic RWW is a triploid organism and its distribution range is much larger than in sexually reproducing populations. We provide valuable molecular evidence for parthenogenesis and analyze the cellular basic process of parthenogenetic RWW. Results show in particular that the parthenogenetic RWW has chromosome unipolar division during meiosis, *Tws* (a regulatory subunit of protein phosphatase 2A) has low expression in ovarian tissue, and *REC8* (paralogous gene of *Verthandi*/*Rad21*) is normally expressed. Our study indicates that parthenogenetic RWW produces triploid egg cells via chromosome unipolar division, while low expression of *Tws* within ovarian tissues may be associated with parthenogenesis, thus providing the first molecular genetic evidence for the parthenogenetic RWW.

**Abstract:**

Rice water weevil (RWW) is divided into two types of population, triploid parthenogenesis and diploid bisexual reproduction. In this study, we explored the meiosis of triploid parthenogenesis RWW (Shangzhuang Town, Haidian District, Beijing, China) by marking the chromosomes and microtubules of parthenogenetic RWW oocytes via immunostaining. The immunostaining results show that there is a canonical meiotic spindle formed in the triploid parthenogenetic RWW oocytes, but chromosomes segregate at only one pole, which means that there is a chromosomal unipolar division during the oogenesis of the parthenogenetic RWW. Furthermore, we cloned the conserved sequences of parthenogenetic RWW *REC8* and *Tws*, and designed primers based on the parthenogenetic RWW sequence to detect expression patterns by quantitative PCR (Q-PCR). Q-PCR results indicate that the expression of *REC8* and *Tws* in ovarian tissue of bisexual *Drosophila melanogaster* is 0.98 and 10,000.00 times parthenogenetic RWW, respectively (*p* < 0.01). The results show that *Tws* had low expression in parthenogenetic RWW ovarian tissue, and *REC8* was expressed normally. Our study suggests that the chromosomal unipolar division and deletion of *Tws* may cause parthenogenesis in RWW.

## 1. Introduction

Parthenogenesis is a reproductive mode in which female animals produce offspring from unfertilized eggs [1]. It has been reported that about 600 species of beetles in 20 families in Coleoptera are able to produce offspring by parthenogenesis [2,3]. While parthenogenesis exists in many taxa, the molecular mechanism and meiotic process of parthenogenesis are still poorly understood. Rice water weevil (RWW), one of the main pests of rice (*Oryza sativa* L.), is native to North America and widely distributed in the southeastern United States [4]. The parthenogenetic RWW was first discovered in California in 1959 [5]. In 1978, RWW was discovered in Aichi Prefecture Japan, and was identified as a triploid parthenogenetic population via karyotype analysis; it then spread to South Korea and China [6,7]. In 2005, it was reported that the parthenogenetic RWW was also found in Italy [8]. Currently, bisexual RWWs only exist in North America [9], while the parthenogenetic form is widely distributed in various countries, showing stronger environmental adaptability and invasive ability [10]. Previous studies have distinguished the chromosome ploidy of RWW between bisexual reproduction and parthenogenesis (2 n = 22 and 3 n = 33, respectively) [6,11], and suggested that both chromosome polyploidization and parthenogenesis could increase the environmental adaptability and invasiveness of organisms [12,13]. Here, we studied the meiotic process, the expression pattern of *REC8* (a paralogous gene of *Verthandi*/*Rad21* in insects, connecting homologous chromatid to maintain synaptic complex structures) and *Tws* (encoding a regulatory subunit of protein phosphatase 2A) gene in parthenogenetic RWW ovarian tissues, providing important insight for further study of the cellular and molecular mechanisms in parthenogenetic organisms.

In comparison to bisexual reproduction, little is known about the molecular mechanism and meiotic process of parthenogenesis; in particular, there is little research concerning the triploid parthenogenetic RWW. The most critical part of parthenogenesis is that the unfertilized oocyte lacks the chromosome set from sperm. Parthenogenetic organisms adopt various tactics to restore parental chromosome number in the oocyte to ensure normal development [14,15] (pp. 63–74); that can be reconciled with two main strategies: Changing the process of meiosis and automixis. In Rickettsia-infected *Neochrysocharis formosa* (Westwood) parasitoids, only a single equational division followed by the expulsion of a single polar body takes place [16]. On the other hand, some lineages of *Daphnia pulex* produce diploid resting eggs instead of meiosis via a mechanism that is genetically equivalent to mitosis [17,18]. Parthenogenetic *Periplaneta americana* [19], *Drosophila mercatorum* [20], *Reticulitermes speratus* [21], *Extatosoma tiaratum* [22], *Drosophila albomicans* [23], *Periplaneta americana* [19] and *Bombyx mori* [24] restore chromosome ploidy by automixis of oocyte. However, it is not clear so far how the triploid parthenogenetic RWW oocytes restore chromosome ploidy and chromosome behavior during meiosis.

In meiosis, through two consecutive cell divisions, haploid germ cells are produced from a diploid chromosomal parent cell. The chromosomes are subject to strict regulative processes during the two divisions to ensure accurate separation into progeny cells (the homologous chromosomes in meiosis I and the sister chromatids in meiosis II). In meiosis, chromosome behavior not only depends on centromere dynamics but it is also linked to the temporal and regional control of sister chromatid cohesion [25,26,27]. At the metaphase of meiosis I, homologous chromosomes are paired through synaptic complexes, and sister centromeres in each homologous chromosome are restricted as functional units [26]. At the anaphase of meiosis I, when the spindle tries to pull the homologous chromosomes to the opposite pole, the cohesion between the sister chromatids keeps the homologous chromosomes together. This mechanism triggers the mechanism of homologous separation [28]. *REC8* encodes meiosis-specific cluster protein subunits that comprise SMC (chromosome structural maintenance), which provides cohesion between sister chromatid arms during meiosis [29]. SMC is cleaved by an endopeptidase called Separase during the metaphase to anaphase transition period [30]. During meiosis I, Shugoshin protein (Sgo) recruits protein phosphatase 2A *(PP2A*)/*mts*-*Tws* in the centromeric region, thus keeping *REC8* locally in a cleavage-resistant unphosphorylated state [31,32,33]. Consequently, sister centromeres remain paired throughout meiosis I and the second burst of separase activity destroys pericentromeric cohesion before anaphase meiosis II to permit their bi-orientation during meiosis II. [34,35]. The synaptic complexes of triploid organisms generally coexist as trivalent and bivalent during meiosis I [36]. The instability of chromosome pairing in meiosis I metaphase leads to their random separation, which often results in a high incidence of miscarriage and easy reproductive isolation. Therefore, triploidy is considered the end of biological evolution to a certain extent [37]. Polyploid organisms can achieve stable meiotic behavior and genome stability only after a mechanism to ensure homologous chromosome recombination and separation is established [37]. Rice with *REC8* mutation can produce unfertilized diploid oocytes [38]. Parthenogenetic *Daphnia pulex* carries a *REC8* allele containing a 1239 bp element into a 604 bp site upstream of a transposable element as well as a frameshift mutation, both of which are completely absent from sexual lineages [39]. The GB45239 gene, which encodes the SMC protein, is almost not expressed in the ovaries of parthenogenetic honeybees (*Apis mellifera capensis*) [40]. Knockdown of the maternal PP2A catalytic subunit in *Oikopleura dioica* caused unfertilized eggs to spontaneously release polar bodies after spawning, and then start pseudo-cleavages without fertilization, namely, parthenogenesis [41]. We want to explore whether the *REC8* and *PP2A*-*Tws* are also involved in the parthenogenesis of the RWW.

In this study, we demonstrate the meiosis process of the parthenogenetic RWW and detect the expression patterns of *REC8* and *Tws* in ovarian tissues. Our research found that the chromosomes of the triploid parthenogenetic RWW segregate at one pole of the meiotic spindle in the anaphase of meiosis I. Chromosomal unipolar division is a new strategy to restore chromosomal numbers in parthenogenetic species. The expression of *REC8* in RWW ovarian tissue is normal, but *Tws* is almost not expressed. Therefore, we recognize the absence of *Tws* and chromosomal unipolar division may account for RWW parthenogenesis.

## 2. Materials and Methods

### 2.1. Rice Water Weevil and Fly Stocks

The parthenogenetic RWW was collected at Shangzhuang Town, Haidian District, Beijing (40.1125 N, 116.1953 E), China, in June 2019. The abdomen was immediately dissected to obtain ovarian tissue, immunostaining experiments were performed immediately and the remaining tissues were stored in liquid nitrogen for RNA extraction. Flies were raised in a standard cornmeal molasses medium at 25 °C, 60% humidity in a 12 h:12 h light:dark cycle. The ISOCS (Wild-type *Drosophila melanogaster*) was a gift from Professor Chuan Zhou (Institute of Zoology, Chinese Academy of Sciences).

### 2.2. Immunostaining

Ovarian tissue fixation was performed according to standard methods [42,43]. The parthenogenetic RWW ovarian tissue was fixed with a solution containing heptane (200 μL of PBS containing 2% EM-grade formaldehyde and 0.5% Nonidet P-40, mixed with 600 μL heptane). The ovaries were incubated in the primary antibody at 4 °C for 12 h. Primary and secondary antibodies were diluted in PBS (21-040-CVR, Corning, Glendale, AZ, USA) containing 1.5% BSA, 0.3% Tween-20 and 0.2% Triton X-100. Fluorescent images were acquired using a Carl Zeiss LSM710 confocal microscope (Carl Zeiss, Oberkochen, Germany).

The antibody used for immunostaining is Rat anti-α-Tubulin (1:100, MAB1864, Sigma-Aldrich, Burlington, MA, USA). The concentration of DAPI (C0065, Solarbio Life Science, Beijing, China) is 0.5 μg/mL. For detailed experimental procedures, please refer to Supplementary Table S1—Reagent and immunostaining experimental procedure.

### 2.3. Total RNA Extraction and Reverse Transcription

Total RNA was isolated from the collected ovarian tissue using Trizol reagent (Invitrogen Life Technologies, Carlsbad, CA, USA) according to the manufacturer’s instructions. Samples were treated with RNase-free DNase (6215B, Takara Biotechnology Co. Ltd., Dalian, China) to remove any traces of genomic DNA. We measured the purity and concentration of total RNA at 260 nm and 280 nm with a spectrophotometer (Nanodrop 2000, Thermo Fisher Scientific, Shanghai, China). The absorbance (260/280 nm) of all samples was between 1.8 and 2.0. Then, 1 µg of RNA was used for reverse transcription to obtain cDNA (6215 B, Takara Biotechnology Co. Ltd., Dalian, China), following the Takara instructions.

### 2.4. Gene Cloning and Q-PCR

Primers for cloning the *REC8* and *Tws* genes were designed on the sequences of the rice weevil *Sitophilus oryzae* (*REC8*: XM_030908656.1; *Tws*: XM_030903493.1) and the mountain pine beetle *Dendroctonus ponderosae* (*REC8*: XM_019917130.1; *Tws*: XM_019916086.1). PCR conditions were according to the manufacturer’s protocol (RR001, Takara Biotechnology Co. Ltd., Dalian, China). Using cDNA as a template for PCR, the obtained nucleic acid was ligated to pMD18-T vector (6011, Takara Biotechnology Co. Ltd., Dalian, China) using TA cloning technology and sequenced by BGI (Shenzhen, China). The sequences were analyzed by BLAST (BLAST+ 2.3.0 NCBI).

Total RNA was re-extracted from the RWW ovarian tube and reverse transcribed to synthesize cDNA. Q-PCR was performed in StepOnePlus^TM^ Real-time PCR system (Thermo Fisher Scientific, Shanghai, China). TB Green *Premix Ex Taq* kits (RR420, Takara Biotechnology, Dalian, China) were used for Q-PCR with the internal control of *β-actin*. Data were analyzed with the classic 2^−ΔΔCt^ method. All primers were designed and synthetized by BGI (Beijing, China), and the primer sequences are given in Supplementary Table S2—gene cloning and Q-PCR primer sequence—and Supplementary Table S3—gene cloning and Q-PCR experimental procedure.

### 2.5. Statistical Analysis

The Q-PCR results are presented as mean ± standard error of the mean (SEM) based on at least three independent experiments. Statistical test (i.e., one-way analysis of variance (ANOVA)) for all data, as well as results visualization, were performed using Prism 8 (v8.3.1) software. *p* < 0.05 was considered as a statistically significant difference among means.

## 3. Results

### 3.1. The Chromosomes of Parthenogenetic RWW Segregate at One Pole of the Meiotic Spindle during the Meiosis I Anaphase

**Figure 1:**
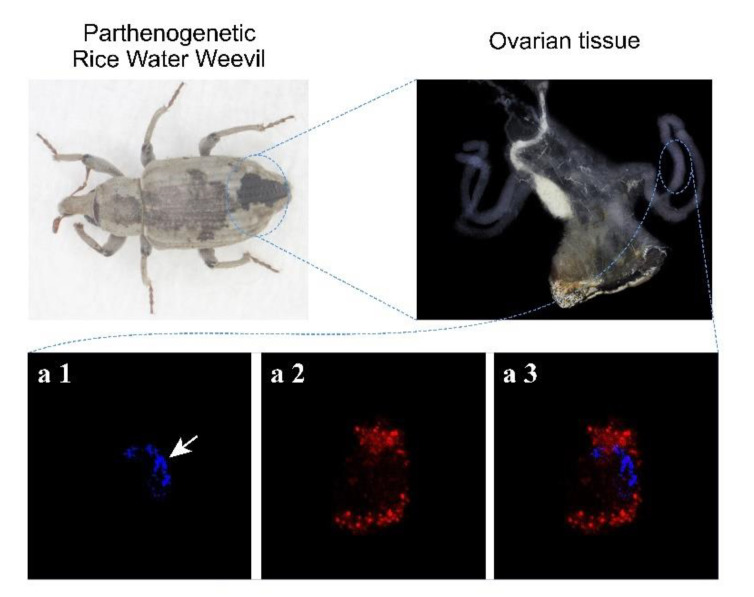
Observation of parthenogenetic ovarian tissue staining. Chromosomes and microtubule organization in rice water weevil (RWW) oocyte were stained with DAPI (white arrow, **a1**) and anti-Tubulin antibodies (in red, **a2**), respectively. Chromosomes move to one end of the cell during the meiosis I anaphase (**a3**).

**Figure 2:**
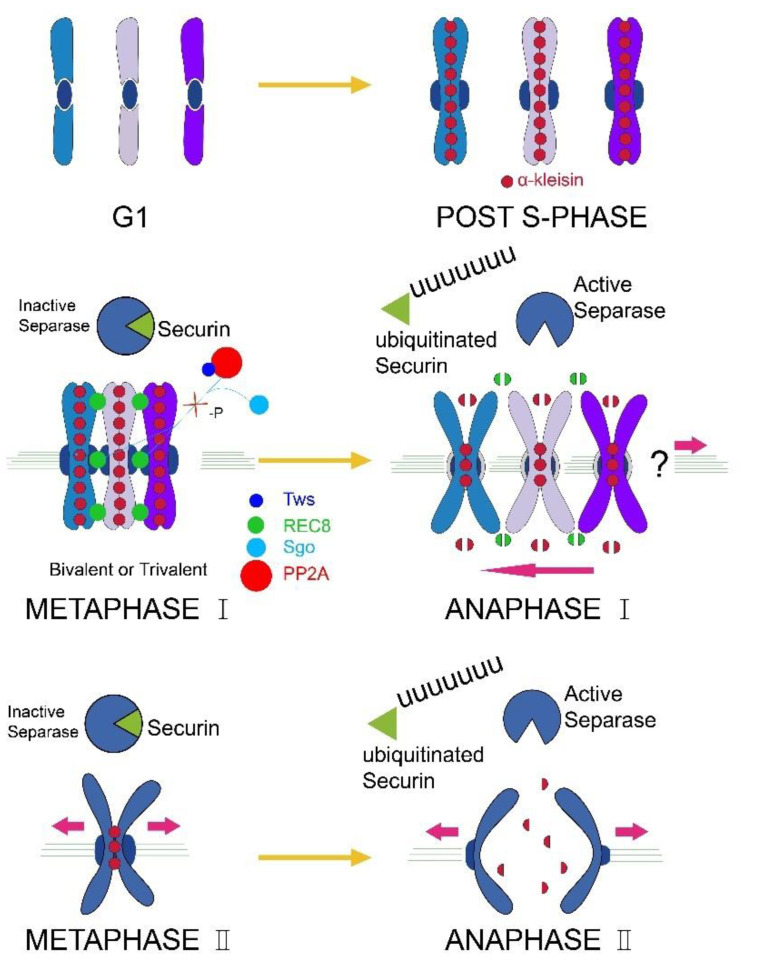
Triploid parthenogenetic RWW meiotic pattern diagram. Parthenogenetic RWW has three chromosome sets (G1). In S phase, chromosome duplication (POST S-PHASE). The low expression of *Tws* prevents the PP2A protein from maintaining the low phosphorylation state of the chromosome (METAPHASE I), which causes the REC8 protein to be cleaved by Separase earlier. All chromosomes segregate at one pole of the meiotic spindle during the anaphase of meiosis I (ANAPHASE I). Subsequently, the oocyte undergoes a standard meiosis II to produce triploid eggs (METAPHASE II and ANAPHASE II).

RWW has a telotrophic type ovary; thus, we dissected the ovary of parthenogenetic RWW and individual ovarian tube tissues were immunostained for microtubules and chromosomes. The results showed that a canonical meiotic spindle is formed, while chromosomes segregate at only one pole of the anaphase cell, and there is no chromosome on the other pole (Figure 1). The white arrow in Figure 1 refers to the chromosomes moving towards one pole of the spindle. This result suggests that parthenogenesis RWW produces triploid eggs via chromosome unipolar division. The meiotic process of parthenogenetic RWW (Figure 2) therefore involves unipolar segregation of all chromosomes during anaphase I followed by a traditional meiotic second division leading to the production of triploid eggs.

### 3.2. Tws and REC8 Expression Levels

**Figure 3:**
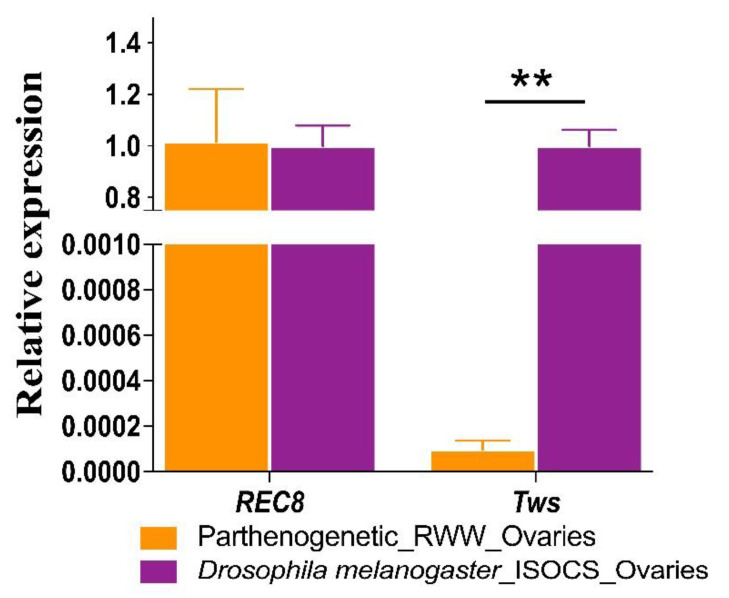
Quantification of *REC8* and *Tws* in RWW ovarian tissues with bisexual reproductive ISOCS as reference. The *y*-axis indicates the relative expression (for details see Table 1, *β-actin* as internal reference). Error bars are s.d., n = 3. ** *p* < 0.01 (*t*-test).

To explore the genes that cause meiosis I anaphase chromosomal behavior abnormalities in parthenogenetic RWW, we cloned the conservative sequences of parthenogenetic RWW *REC8* and *Tws* genes, and the GeneBank accession number: *REC8*: MW560171; *Tws*: MW560172. The *REC8* and *Tws* sequences are given in Supplementary Figure S1—Sequences of gene cloning. To investigate whether the parthenogenesis of RWW is related to these genes, we detected the expression levels of *REC8* and *Tws* by Q-PCR. The Q-PCR results showed that the expressions of *REC8* and *Tws* in ovarian tissues of bisexual *Drosophila melanogaster* (ISOCS) are 0.98 (1.00/1.02 ≈ 0.98) and 10,000.00 (1.00/9.07 × 10^–5^ ≈ 10,000.00) times parthenogenetic RWW, respectively (*p* < 0.01). The results showed that the expression pattern of *REC8* in the ovarian tubes of parthenogenetic RWW was the same as for sexual reproductive flies, and the expression of *Tws* was extremely low (*p* < 0.01; Figure 3, Table 1). The results indicate that PP2A-Tws cannot maintain the dephosphorylation state of the REC8 protein due to the low expression of *Tws* in the ovarian tissue of parthenogenetic RWW. Therefore, REC8 is cleaved by the isolating enzyme in the anaphase of meiosis I. We speculated that the low expression of the *Tws* may be one of the reasons for the parthenogenesis of the RWW.

## 4. Discussion

Parthenogenesis is a research hotspot in the field of developmental biology and plays an important role in the evolution of insect sexuality and reproductive strategies. The triploid parthenogenetic RWW has chromosomal unipolar division during oogenesis. It is known that the RWW has a telotrophic type of ovary [11], and only the cells in the meiosis I have nutritive cord [44,45]. According to the results of immunostaining and transmission electron microscope investigation, the anaphase meiotic I oocytes with nutritive cord only have chromosomes at one pole of the cell [10,44,45,46], which is consistent with our experimental results. It is confirmed that chromosomal unipolar division occurs in meiosis I. The parthenogenetic RWW maintains the parental chromosome number via a modification of the meiotic process of meiosis, a novel strategy different from the eulophid parasitoid *Neochrysocharis formosa* [15,16] and rice [38]; these meiotic cells undergo only a single equational division. This study also found that the *Tws* has low expression in the ovarian tissue of the parthenogenetic RWW, which may be one of the reasons for the parthenogenesis of the RWW. Nonetheless, the molecular mechanism that causes the unipolar division of chromosomes and the molecular mechanism of how the downstream genes controlled by the *Tws* cause parthenogenesis remain to be studied.

There are various reasons and methods for biological parthenogenesis. In ploidy restoration of parthenogenetic animals, meiosis is either preserved in some form (automixis) or is largely suppressed (parthenogenesis) [14]. Obligate parthenogenesis RWW is a triploid organism, which is different from silkworms (*Bombyx mori*) and aphids (*Acyrthosiphum pisum*) [24,47,48,49,50,51,52,53,54] [55] (pp. 199–257) whose reproductive strategies can be changed by environmental factors. Moreover, parthenogenetic RWW does not require sperm to participate in the entire life cycle, which is different from nematodes (*Mesorhabditis belari*) [56] that require sperm to activate unfertilized eggs for parthenogenesis. These results imply that diploid parthenogenetic organisms retain the complete meiotic process and meiotic genes [20,53]. In triploid parthenogenetic male *Cacopsylla myrtilli* meiosis, all univalent chromosomes divide during the first meiotic division resulting in the incomplete second meiotic division and formation of diploid sperms [57,58,59]. These studies collectively show that triploid parthenogenetic species are prone to modify meiosis processes, so genes related to meiosis will also change. In this study, immunostaining technology was used to observe the behavior of chromosomes and microtubules during the first division of parthenogenetic RWW meiosis. Our results show that the parthenogenetic RWW restored the parental chromosome number via chromosome unipolar split division, which is different from other parthenogenetic species. With the improvement of insect egg cell culture technology in vitro, the entire process of meiosis can be tracked and observed, which can better explain the cytological mechanism of parthenogenetic insects [60].

In meiosis, chromosomes are controlled by the behavior of the centromere and the time and area of sister chromatid cohesion during the two divisions to ensure accurate separation into daughter cells [25,26]. A unique proteic structure, the synaptonemal complex, is formed during the early stages of prophase I between the homologs [61,62]. The *Drosophila melanogaster* with *Rad21* (*REC8* homologous gene) mutation produces diploid unfertilized eggs (1/83), indicating that *REC8* may be related to the chromosome accurate separation of female meiosis I [27]. Levels of REC8 protein in *Tws* mutant individual were reduced to about 50% compared with the wild type, which indicates that *PP2A* is required mainly to maintain the level of REC8 protein but not *REC8* mRNA expression [31]. *PP2A* is composed of a trimeric core enzyme composed of structural A, catalytic C and regulatory B subunits [63], and plays a very important role in many physiological processes, especially in cell division [64]. Mutations in both *Tws* and *PP2A* can cause abnormal spindle assembly, resulting in an abnormal chromosomal arrangement, but the chromosomes can be separated normally to the two poles of the cell [32,65]. These results indicate that *PP2A-Tws* and *REC8* can maintain the stability of the chromosome structure during meiosis. In *Drosophila melanogaster* cells, PP2A-Tws promotes nuclear envelope reformation during mitotic exit by dephosphorylating barrier-to-autointegration factor [66]. In *Oikopleura dioica* the knockdown of the maternal PP2A catalytic subunit caused unfertilized eggs to spontaneously release polar bodies after spawning, and then start pseudo-cleavages without fertilization, namely, parthenogenesis. On the other hand, egg cells with completely inactivated *PP2A* have aberrations in cleavage and cannot develop into a new individual [40]. These results illustrate that *PP2A-Tws* play an important role in the process of meiosis, and the complete inactivation of *PP2A* is detrimental to egg development. In parthenogenetic RWW, *Tws* and *REC8* expression levels suggest that *Tws* can cause REC8 protein to be cleaved prematurely, which may be the cause of chromosome unipolar division. When *Tws* has low expression, some maternal proteins could be over-phosphorylated, which may lead to parthenogenesis of RWW.

In summary, we find that all chromosomes of parthenogenetic RWW segregate at one pole of the meiotic spindle in meiosis I anaphase and the *Tws* in the parthenogenetic RWW ovarian tube tissue had low expression. We conclude that the chromosome unipolar division and low expression of *Tws* together caused parthenogenesis of RWW. Triploid parthenogenesis in RWW shows a new way of meiosis, but it also confirms that parthenogenesis is prone to change in the biological meiosis process. It has been reported that *Tws* is the regulatory subunit of PP2A, which not only plays an important role in meiosis but also participates in the Wg/Wnt signaling pathway to regulate the development of *Drosophila* wings [67,68,69]. While we have elucidated the meiosis process of triploid parthenogenesis rice water weevil, the relationship between *Tws* and chromosome monopolar division has not been found yet. Our study opens an avenue to elucidate the unique chromosomal segregation mechanism of meiosis for parthenogenetic organisms. The specific function of *PP2A-Tws* needs to be further studied to unravel the relationship between *Tws* and parthenogenesis.

## Figures and Tables

**Table 1:** Q-PCR results.

Species	Relative Expression	
	*REC8*	*Tws*
*Lissorhoptrus oryzophilus-P * ^1^	1.02 ± 0.20	9.70 × 10^−5^ ± 0.4 × 10^−5^
*Drosophila melanogaster*	1.00 ± 0.08	1.00 ± 0.0627

^1^*Lissorhoptrus oryzophilus-P*: Parthenogenetic RWW. All data retain three significant figures; each experiment was repeated three times (n = 3). The relative gene expression level is based on the standardized bisexual reproduction of *Drosophila melanogaster* (ISOCS) as a reference.

## Data Availability

All data generated or analyzed during this study are included in this published article.

## Supplementary Materials

The following are available online at https://www.mdpi.com/2075-4450/12/4/278/s1. Table S1: Reagent and Immunostaining experimental procedure, Table S2: Gene cloning and Q-PCR primer sequence, Table S3: Gene cloning and Q-PCR experimental procedure, Figure S1 Sequences of gene cloning.
