# Supplementary material for: Chromosome Unipolar Division and Low Expression of Tws May Cause Parthenogenesis of Rice Water Weevil (Lissorhoptrus oryzophilus Kuschel)
Source: Insects. 2021 Mar 24;12(4):278. doi: 10.3390/insects12040278 (PMC8064085; doi:10.3390/insects12040278)
Supplement: Supplementary file 1 [file insects-12-00278-s001.zip › Supplementary Material/Table S1.pdf]

Supplemental Information

Table S1 Reagent and Immunostaining experimental procedure

RWW ovarian tissue immunostaining experimental procedure

1. Reagent formula

Formaldehyde/Heptane fixed buffer (800 μ L)

Reagent	Dosage
Nonodet P-40	1 μL
Formaldehyde	4 μL
1×PBS	195 μL
Heptane	600 μL
Total	800 μL

PBTA (20 ml)：

Reagent	Dosage	Final concentration
10×PBS	2 mL	1×
30% Tween-20	200 μL	0.1~0.3%
20%Triton X-100	200 μL	0.2%
BSA	2 mL	1.5%
dd H_2O	Up to 20 mL	

PBT (100 ml)：

Reagent	Dosage	Final concentration
10×PBS	10 mL	1×
30%Tween-20	1 mL	0.1~0.3%
20%Triton X-100	1 mL	0.2%
ddH_2O	Up to 100 mL	

Mounting media (500 μL):

Reagant	Dosage
10×PBS	50 μL
Glycerol	200 μL
ddH_2O	250 μL
Total	500 μL

2. Steps:

(1) Anesthetize RWW with CO_2 and dissect the ovarian tissue in 1×PBS, immediately, fixed in formaldehyde/Heptane fixed buffer on shaker (100 rpm; TS-2, Haimen Kylin-Bell Lab Instruments Co., Ltd. Jiangsu, China) for 30 mins;

(2) At room temperature, the ovaries are washed four times on PBT at 100 rpm for 15 minutes each time;

(3) The ovaries blocked overnight in 4 ℃, PBTA on a shaker at 100 rpm;

(4) Repeat step (2), then wash with PBT for 1.5 hours;

(5) The ovaries incubated 12 hours in α-Tubulin antibody diluted in PBTA, 4 ℃, shaker at 100 rpm;

(6) Discard primary antibody, then repeat step (2);

(7) The ovaries were incubated in appropriate secondary antibody and DAPI diluted in PBTA for 4 hours, 4℃, shaker at 100 rpm, dark;

(8) Repeat step (2);

(9) Take a clean glass slide, drop 20 μL of mounting media in the middle, and add the ovaries to the mounting media. Dissect the upper part of the ovarian tissue under a stereoscope, remove excess tissue, and cover with a coverslip. Apply nail polish around the coverslip;

(10) Take pictures immediately with a fluorescent confocal microscope.
