# Supplementary material for: Chromosome Unipolar Division and Low Expression of Tws May Cause Parthenogenesis of Rice Water Weevil (Lissorhoptrus oryzophilus Kuschel)
Source: Insects. 2021 Mar 24;12(4):278. doi: 10.3390/insects12040278 (PMC8064085; doi:10.3390/insects12040278)
Supplement: Supplementary file 1 [file insects-12-00278-s001.zip › Supplementary Material/Table S2.pdf]

Supplemental Information

Table S2 Gene cloning and Q-PCR primer sequence

Primer Name	Sequence (5'-3')
	For Gene Clone
REC8	F: TTATTCCCATTTTATTTTGGC<br>R: ACAAAGGCTTCATTGCAATC
Tws	F: TTCGACAAATTCGAGTGCTG<br>R: ATCTAAACAGTCCACACTGAT
	For Q-PCR
*REC8*_Q_RWW	F: TTGAGACTTCTGTTGATGGT<br>R: AGCAGATACTTAGCCTTTCG
*Tws*_Q_RWW	F: GCTACAACAACTTCCTCCG<br>R: CACACTGATCTCATCCTTCT
*REC8*_Q_*Drosophila melanogaster*	F: AAGAGAACGAGAACGCATT<br>R: GATTCGCTCTCAGGACTTC
Tws _Q_*Drosophila melanogaster*	F: ATCAGCGTGGACTGTCTA<br>R: GGTTCTTGTGAGGAGTGTT
*β-actin*_Q_RWW	F: AAACACTGTCCTCTCTGG<br>R: GAGTATTTCCTTTCTGGG
*β-actin*_Q_ *Drosophila melanogaster*	F: CCAACCGTGAGAAGATGACC<br>R: GCCGGAGTCCAGAACGATAC

The *REC8* and *Tws* primers were used for gene cloning; *REC8*_Q_RWW, *Tws*_Q_RWW, *REC8*_Q_*Drosophila melanogaster*, *Tw* _Q_*Drosophila melanogaster*, *β-actin*_Q_RWW, *β-actin*_Q_*Drosophila melanogaster* primers were used for Q-PCR.
