# Supplementary material for: Chromosome Unipolar Division and Low Expression of Tws May Cause Parthenogenesis of Rice Water Weevil (Lissorhoptrus oryzophilus Kuschel)
Source: Insects. 2021 Mar 24;12(4):278. doi: 10.3390/insects12040278 (PMC8064085; doi:10.3390/insects12040278)
Supplement: Supplementary file 1 [file insects-12-00278-s001.zip › Supplementary Material/Table S3.pdf]

Supplemental Information

Table S3 Gene cloning and Q-PCR experimental procedure

Gene cloning and Q-PCR experimental procedure of parthenogenetic RWW.

1. Gene cloning

Use the cDNA obtained by reverse transcription as a template for gene cloning. The reaction process is as follows:

Composition of PCR reaction solution (50 μL)

Reagent	Dosage
TaKaRa *Taq* (5 U/μl)	0.25 μl
10X PCR Buffer (Mg^{2+} plus)	5 μl
dNTP Mixture (each 2.5 mM)	4 μl
Template	1 μg
Upstream primer	1 μM
Downstream primer	1 μM
ddH_2O	Up to 50 μl

Reaction conditions:

Initial denaturation	98 ℃	2min	
Denaturation	98 ℃	10 sec	35 Cycles
Annealing	50 ℃	30 sec	35 Cycles
Extension	72 ℃	30 sec	35 Cycles
Final Extension	72 ℃	10 min	

Purify PCR products according to the kit instructions (AP-GX-50G, Corning, USA), and ligate it to the PMD18-T vector (9057, 6011, Takara Biotechnology Co. Ltd., Dalian, China). Sequencing by BGI (Shenzhen, China).

2. Q-PCR

Real-time quantitative PCR was performed in the StepOnePlus™ Real-time PCR system (Thermo Fisher Scientific, Shanghai, China). TB Green Premix Ex Taq kit (RR420, Takara Biotechnology, Dalian, China) was used to for qRT-PCR with the internal control of β-actin.

Reaction conditions:

Initial denaturation	98 ℃	2min	
Denaturation	98 ℃	10 sec	35 Cycles
Annealing	49 ℃	30 sec	
Extension	72 ℃	10 sec	
Final Extension	72 ℃	10 min	

After each extension, detect the absorbance value.

Data were analyzed by the classic $2^{-\Delta\Delta Ct}$ method.
